# Physical and Mental Health Factors Associated with Poor Nutrition in Elderly Cancer Survivors: Insights from a Nationwide Survey

**DOI:** 10.3390/ijerph18179313

**Published:** 2021-09-03

**Authors:** Mikyong Byun, Eunjung Kim, Jieun Kim

**Affiliations:** 1 BK21FOUR R&E Center for Learning Health Systems, College of Nursing, Korea University, Anam-dong, Seongbuk-gu, Seoul 02841, Korea; mulanbb@korea.ac.kr; 2 Department of Nursing Science, Catholic Kwandong University College of Medicine, 24 Beomil-ro 579beon-gil, Gangneung-si 25601, Korea; 3 Department of Nursing, Dongyang University, 145, Dongyangdae-ro, Punggi-eup, Youngju-si 36040, Korea; ejissy@gmail.com; 4 Department of Neurology, Gangneung Asan Hospital, University of Ulsan College of Medicine, 38 Bangdong-gil, Sacheon-myeon, Gangneung-si 25440, Korea

**Keywords:** cancer, malnutrition, risk factor, older adults

## Abstract

Elderly cancer survivors (patients with any stage of cancer or a history of cancer) are precious members of our society and they can be easily found in various types of surveys. As is well known, good nutrition is important in elderly people suffering from cancer. Proper nutritional evaluation and intervention not only improves their quality of life but also helps them to receive adequate treatment, thereby prolonging individual survival and reducing social healthcare costs. In this study, we retrieved elderly cancer survivors from national survey data and assessed their nutritional status as good or bad. Then, we described the individual, physical, and mental health factors between people with good and bad nutrition. Physical and psychological variables associated with poor nutritional status were evaluated through regression analysis. We investigated data from the 2017 National Survey of Older Persons, and cancer patients aged 65 years or over were eligible. A total of 360 adults were enrolled and more than half (57.2%, *n* = 206) were in a poor nutritional status. We applied individual variable-adjusted statistical models and discovered that limited instrumental activities of daily living (IADL) (OR 2.15, 95% CI 1.08–4.28) and poor subjective health status (OR 1.74, 95% CI 1.00–3.02) were significantly associated with poor nutrition on logistic regression. Our research findings suggested that IADL and self-rated health status needed to be addressed in old cancer survivors at nutritional risk. The early recognition and management of nutrition in these populations might help them to live longer and have a better quality of life, eventually reducing socioeconomic burdens.

## 1. Introduction

Human life expectancy has increased by three decades over the past century. In this era of global aging, the rapid increase in the numbers of elderly people inevitably prompts personal, social, national, and worldwide distress. The Republic of Korea is now one of the world’s fastest aging countries. It became classed as an ‘aged society’ just 17 years after it was named an ‘aging society’ in 2000 [1]. The proportion of people aged 65 and over reached 15 percent of the total population by the end of 2019. Additionally, an official report from Statistics Korea predicted that South Korea will become the world’s most aged society by 2067 [2].

An overwhelming increment in the aged population has brought a higher prevalence of malignant neoplasms, as well as various degenerative diseases. One in four women and one in three men are diagnosed with cancer during their lifetime, globally [3]. In Korea, cancer has become the top-ranked cause of death over the last ten years, and 229,180 cancer diagnoses were recorded in 2016 alone [4]. In general, risk factors for cancer include age, a personal or family history of cancer, smoking, obesity, alcohol consumption, certain types of infections, and exposure to environmental hazards, etc. [5] Above all things, advanced age is strongly associated with most of the common malignancies such as lung, colon, pancreas, and prostate cancers [6]. By virtue of the advances in public health and medical science, the number of elderly cancer survivors (any adult diagnosed with malignancy) is estimated to grow in an exponential, rather than linear, manner. The natural history of cancer, with a few exceptions, is now being transformed from a lethal disease into a chronic medical condition [7], and this means that malignancy has raised personal interest in quality of life and has become a worldwide healthcare issue [8].

Good nutrition is important for chronic diseases, particularly cancer. Nutrition is a modifiable factor that can reduce the chances of disease progression [9], but up to 85% of cancer patients are at risk of malnutrition during the course of their treatment [10]. Malnutrition in both patients on active treatment and those in survivorship are attributable to various causes: cancer-induced cachexia, metabolic dysregulation, treatment-related toxicities, and psychological causes such as depression, emotional distress, and social isolation [11,12,13]. Poor nutrition hinders old cancer patients from overcoming various morbid conditions, thus resulting in a poor prognosis and survival [13,14]. In other words, adequate nutritional assessment/intervention not only improves the quality of life but also helps them to receive adequate treatment, thereby prolonging individual survival and reducing social healthcare costs.

Cancer survivors are everywhere, living their own lives in our neighborhood. When we pay a little bit of attention to them, they are easily found among various types of surveys. In this study, we hypothesized that we could gain deep insights on nutrition and its related factors in elderly cancer survivors while using large-sized and well-designed data from a national survey. First, we compared individual, physical, and mental health factors between well-nourished and poorly nourished cancer survivor groups using public data. We then aimed to find out statistically significant physical and psychological variables associated with poor nutrition by controlling various individual characteristics.

## 2. Materials and Methods

### 2.1. Data Source

Under the approval of the National Statistical Office, we collected original data from the Korean Health and Welfare Data Portal. Our data derived from the 2017 National Survey of Older Persons (NSOP) [1]. The database was made up of a stratified random sample of about ten thousand adults who were living in general housing facilities, and was designed to be representative of the Korean elderly population. NSOP 2017 took place through in-person interviews, involving 10,299 senior citizens aged 65 or older in 934 survey areas from June to August 2017. The survey was carried out by 60 professional surveyors (separated into 15 teams of four surveyors each, one supervisor in each team), trained by the skilled research staff beforehand.

### 2.2. Patient Selection and Study Design

**Figure 1:**
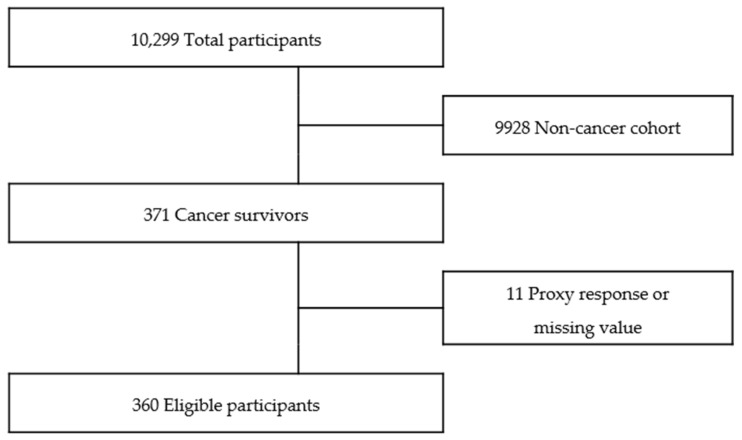
Flow diagram of inclusion of study population.

From the original data of 10,299 participants, a total of 360 cancer survivors with available nutrition status were eligible (Figure 1). Cancer survivors were defined as those who gave an affirmative answer to the following survey item: “Are you suffering from cancer or malignant neoplasm for more than 3 months after doctor’s diagnosis?”

We assessed predictive factors for poor nutrition using three (individual, physical and psychological) pivotal categories based on descriptive, correlational, and cross-sectional design. Afterward, we devised individual variables-adjusted models to determine essential physical and psychological variables in terms of poor nutrition on multiple logistic regression.

### 2.3. Measurements

#### 2.3.1. Nutrition

In accordance with the ‘Determine Your Nutritional Health’ questionnaire of the Nutrition Screening Initiative [15], individual nutritional status was evaluated. This measurement tool was composed of ten binary (“yes” or “no”) items. To each question, a “yes” was scored in a range of 1 to 4, whereas “no” was scored 0. The total scores of ten responses were classified as follows: 0–2: good nutritional state, 3–5: moderate nutritional risk, ≥6: high nutritional risk. Then, a score of 0–2 was considered to be “in good nutrition” while a final score of more than 2 was defined as “in poor nutrition”, as a dichotomy.

#### 2.3.2. Individual Factors

Individual variables were grouped into 4 subcategories: demographic, socioeconomic, health status, and health-related behavior. Firstly, demographic factors were composed of age, sex, and marital status (living with spouse or living without spouse). Secondly, an education level (0–6 yrs, 7–9 yrs, 10–12 yrs, or ≥13 yrs) and degree of household income (Q1 [the lowest quartile], Q2, Q3, Q4, or Q5) of respondents were categorized as socioeconomic variables. Thirdly, chronic medical diseases (hypertension, diabetes, and arthritis), body mass index (BMI), and the number of current medications (0, 1, 2, or ≥3) were classified as health status variables. Lastly, health-related behaviors involved exercise, smoking (past/never or current), and alcohol consumption.

The presence or absence of chronic diseases was assessed based on the following questions: “have you been suffering from any diseases like hypertension, diabetes, mellitus or arthritis for more than 3 months?”, and “have you been diagnosed with the above diseases by any doctor?” Respondents who answered “yes” to both questions were determined to have a chronic disease. Identification of the number of medications was conducted using the next question: “how many prescribed medications have you been taking over the past 3 months or more?”. Exercise for more than 150 min per week is recommended by the World Health Organization (WHO) [16]. A “No” response to the question of “do you usually exercise?” was regarded as “none”. Accordingly, exercise levels were classified into three subtypes: within the recommended level (≥150 min/week), below the recommended level (<150 min/week), and none. Alcohol intake was evaluated using the National Institute on Alcohol Abuse and Alcoholism criteria [17]. In older adults aged 65 years and over, an alcohol intake of less than one standard drink (a 350 mL glass of beer) per day was regarded as a reasonable degree in this study; an intake of more than one standard drink per day was regarded as immoderate. Those who did not drink at all were assigned to “none”.

#### 2.3.3. Physical Health Factors

Physical variables included hearing, vision, activities of daily living (ADL), instrumental activities of daily living (IADL), and muscle strength.

Sensory discomforts were classified as visual and hearing difficulties. Visual discomfort was defined as “uncomfortable” in respondents who answered “uncomfortable” or “very uncomfortable.” Likewise, auditory discomfort was defined as “discomfort” in those who responded “uncomfortable” or “very uncomfortable.”

The degree of ADL limitation was evaluated using the Korean Activities Daily Living scale [18]. This scale consisted of seven domains: “dressing”, “face washing, brushing teeth, and shampooing”, “bathing”, “eating food”, “bowel and bladder control (continence)”, “toilet use”, and “getting up and walking across the room (transfer)”. Each domain was assessed by a 3-point rating method (total independence/partial dependence/total dependence) and a higher score stood for a more severely limited daily living routine.

A score of 0, total independence, was considered as “no limitation”. A score of 1 and 2, indicating “partial and complete dependence”, respectively, was determined as “having limitation”. Individuals who reported any dependency in more than one domain were considered as having an ADL impairment.

Limitation to IADL was assessed according to the Korean Instrumental Activity of Daily Living scale [18]. This scale was composed of ten items: “personal grooming”, “performing household chores”, “preparing meals”, “laundry”, “taking medications on time”, “going out for a short walk”, “shopping”, “managing money”, “ability to make and receive phone calls”, and “using public transportation”. Total independence was classified as “no limitation”. On the other hand, the others (partial, complete, little, much dependence, and cannot be done at all) were regarded as “having limitation”. Additionally, participants who complained of any restriction in more than one item were considered to have an IADL limitation.

The following task, the so-called “five times sit to stand test” requested: “Please sit and rise on a chair or bed with both hands placed in front. Repeat this movement 5 times without using both hands.” This was used for estimating muscular strength. Good muscle strength was defined when tasks were completed without any difficulty. By contrast, either “Tried but couldn’t finish 5 times” or “Can’t even try” was considered as poor muscle strength.

#### 2.3.4. Mental Health Factors

Psychological variables involved insomnia, depression, and subjective health status.

Insomnia was assessed using the following question: “have you been suffering from insomnia for more than 3 months?”

Depression was evaluated based on the Korean version of the 15-item Geriatric Depression Scale (Short-version of GDS-K; SGDS-K) [19]. The optimal cut-off values for SGDS-K in screening clinically meaningful depression were suggested as ≥8 (total scores range from 0 to 15). Based on this proposal, scores of ≥8 and <8 were classified into the dichotomy of “depressed” and “not depressed”.

Self-rated health (SRH) was assessed using the next five-choice Likert scale question, “How would you judge your health status generally?” The five answers were dichotomized into “negative” (very poor or poor) or “positive” (fair, good, or very good) for our analysis.

### 2.4. Statistical Analysis

**Figure 2:**
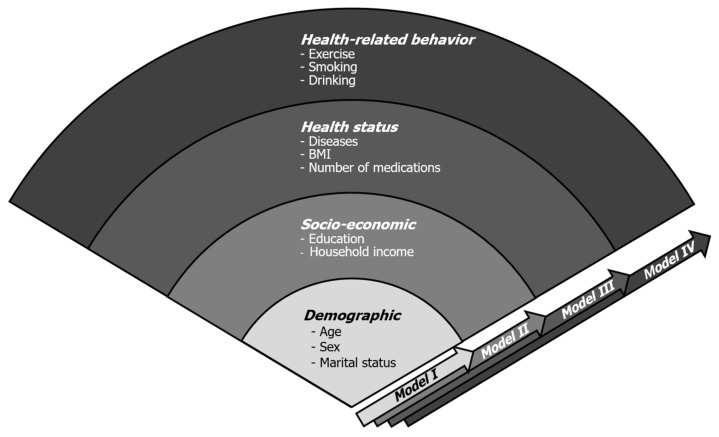
Proposed individual variables-adjusted models. The variables from individual subcategories (demographic, socioeconomic, health status, and health-related behavior) were combined in consecutive order and four experimental models were built.

We performed descriptive statistics using the χ2 or t-test to compare differences in the nutritional status in view of the individual, physical and psychological aspects (*p-value* less than 0.05 is statistically significant). Initially, each independent factor was analyzed for univariate logistic regression, and then, statistically significant factors were selected for carrying out multivariate logistic regression. Afterward, we built our novel individual variables-adjusted models to minimize potentially confounding influences by individual variables. The factors from the individual category, including demographic, socioeconomic, health status, and the health-related behavior subcategories, were combined into groups and entered into logistic regression models step by step. In the process of designing models, we hypothesized that variables in the demographic subcategory (such as age, sex, and marital status) were the least modifiable (depending on one’s effort) among four subcategories. The other subcategories such as socioeconomic, health status, and health-related behaviors could be more easily modifiable in consecutive order. Based on this assumption, we first built Model I (consisting of demographic subcategory only) and then we gradually encompassed easily modifiable subcategories in a stepwise way, which were presented as Model II, III, and IV. (Model I: demographic variables only, Model II: demographic and socioeconomic factors, Model III: demographic, socioeconomic, and health status factors, Model IV: all the individual factors) (Figure 2). Odds ratios (ORs) and the corresponding 95% confidence intervals (CIs) were also presented. The level of statistical significance was set at *p*-value less than 0.05. The data were analyzed using the IBM SPSS version 22.0 software package (IBM, Armonk, NY, USA).

## 3. Results

### 3.1. Prevalence of Poor Nutrition in Elderly Cancer Survivors

Among 360 adults with a history of malignant neoplasm, more than half of them (57.2%, *n* = 206) were in poor nutrition.

### 3.2. Differences in Individual Variables between Good and Poor Nutrition Groups

Differences in nutritional status according to individual variables are summarized in Table 1. With respect to demographic, socioeconomic, health status, and health-related behavior components, about half of the items (age, sex, marital status, hypertension, arthritis, and the number of medications) showed statistical significance between good and poor nutrition groups.

### 3.3. Differences in Physical and Psychological Variables between Good and Poor Nutrition Groups

Differences in nutritional status according to physical and psychological variables are summarized in Table 2. ADL and IADL limitations, as well as poor muscle strength, were more frequently observed in the poor nutrition group. Likewise, statistically significant differences were found between the two groups in terms of physical factors such as ADL, IADL, and muscle strength.

There were also statistically significant differences between the two groups in view of mental health factors such as insomnia, depression, and subjective health status (Table 2).

### 3.4. Multivariable Logistic Regression Analysis of Factors in Elderly Cancer Survivors with Poor Nutrition Using Individual Variables-Adjusted Models

To evaluate the physical and psychological factors affecting risks for malnutrition, we applied our unique individual variable-adjusted models, as mentioned above. In Model IV, poor nutrition in elderly cancer survivors was significantly associated with IADL limitation (odds ratio (OR) 2.15, 95% confidence interval (CI) 1.08–4.28) and negative SRH (OR 1.74, 95% CI 1.02–3.02) on multivariate analysis (Table 3). Remarkably, both variables were consistently meaningful from Model I to IV.

## 4. Discussion

### 4.1. Nutrition Is Significant in Old Cancer Survivors but Is Overlooked or Undermanaged in the Real World

Nutrition is significant in senior cancer patients in terms of quality of life and even survival, but, in reality, malnutrition is very common among cancer survivors. To make this situation worse, it is readily overlooked or undermanaged even after it is detected by healthcare providers [20]. Most cancer survivors are highly motivated to seek potentially beneficial nutritional ingredients or behaviors for themselves [21], but healthcare professionals have little or no interest and sometimes simply refer patients to nutritional counselors. Poor nutrition cannot be overemphasized because it results in weight loss, sarcopenia, immune deficiencies, infections, vulnerability to stressors, and higher mortality [7]. This is why proper nutritional assessment and timely intervention are needed for elderly cancer survivors. Maintaining good nutritional status not only improves the individual quality of life but also helps to increase treatment compliance. Nutritional support helps cancer survivors to live longer and healthier, eventually reducing socioeconomic healthcare burdens on society.

Not to mention the problem lists on the medical record, healthcare workers need to pay additional attention to patients’ individual, physical, and psychological characteristics. Of course, this takes considerable time and effort for medical staff, but cancer patients deserve it. Given the importance of nutrition during the treatment and surveillance phase, it is a worthwhile effort to explore meaningful factors associated with nutrition. Furthermore, cancer survivors are everywhere and easily identifiable in various types of public or private surveys. In this regard, insights from national survey data (which is primarily based on comprehensive face-to-face interviews) might be more feasible than those from disease-focused medical investigations in certain situations like cancer-related malnutrition.

### 4.2. Old Cancer Survivors Are at Risk of Malnutrition If They Complain of IADL Limitation or If They Feel Unhealthy

In this study, we found that both IADL and SRH statuses were statistically associated with nutritional health in old survivors. We have to keep in mind that patients are at risk of malnutrition if they complain of any limitation to IADL or if they perceive themselves as unhealthy.

The ADL refers to a constellation of fundamental activities required to accomplish independence as an individual member of society [22]. Elderly cancer survivors frequently complain about an incessant physical burden, exercise intolerance, and general deconditioning. All of these can negatively affect their ability to perform independent daily routines [23]. The ADL can be divided into two subcategories: physical ADL (PADL) and instrumental ADL (IADL) [24]. The PADL encompasses basic care activities like personal hygiene, feeding, toileting, ambulating, and dressing, while IADL refers to more complex tasks such as shopping, meal preparation, using transportation, doing household chores, and managing medications. Compared to PADL, IADL requires more organizational thinking skills [25]. Therefore, IADL might be more easily influenced by cognitive or mental changes along with decreased physical ability. Thus, the IADL needs to be assessed and considered as a different concept from ADL [26]. Even in the case of seemingly healthy old people managing themselves well, there might be subtle impairments in the IADL. For example, in the early course of anti-cancer treatment, patients have more trouble with IADL than with PADL. This reflects the fact that components of IADL need a somewhat higher level of functional capabilities [27,28]. Another systematic review also demonstrated that one-half required assistance to perform IADL among 19,246 adult cancer patients aged over 18 [22]. Based on these findings, we can forecast that IADL limitations could be more common and serious for those old cancer survivors.

There is growing evidence of poor SRH and its relationship with future morbidity and mortality [29,30]. Unfortunately, the same goes for cancer survivors. Shadbolt et al. reported that SRH status could be a predictor of survival in advanced-stage cancer patients [31]. Certainly, one of the most important objectives of cancer treatment is to prolong one’s life expectancy. But treatment processes are often accompanied by intractable cancer pain, sustained fatigue, reduced physical fitness, and psychiatric problems such as depression [32,33]. Previous studies proposed an association between poor nutrition and negative SRH in general and in hospitalized populations [34,35]. Hospitalized patients who regarded them as unhealthy had a tendency to have an insufficient food intake, which increased the risk of in-hospital mortality.

### 4.3. Limitations and Strengths of Our Study

As mentioned above, our study revealed IADL limitations (physical health factor) and poor SRH (mental health factor) are in association with malnutrition risk. Actually, both IADL and SRH are multidimensional concepts per se; a variety of individual, physical, and psychological factors positively or negatively affect them. Thus, it is usually advised to interpret IADL and SRH from a holistic perspective. To improve cancer patients’ nutritional statuses, limited IADL and negative SRH must be recognized as early as possible to provide well-timed nutritional management. Good nutrition extends cancer-related survival and alleviates social healthcare burdens in the long run.

There were three major limitations in this study. First, our data analysis had a cross-sectional design, which may not always find causality; the cause-and-effect relationship between cancer and malnutrition could be ambiguous, which was an inherent limitation of the study design. Second, there may be a possible recall bias as the primary data source was interview responses. Third, specific cancer location, type, stage, duration since diagnosis, and treatment status were not available since this official survey focused on the general elderly population rather than on cancer patients. In addition, only survivors diagnosed with cancer for more than 3 months were eligible, while the other “within 3 months” survivors were left unanalyzed. A major disadvantage of using secondary data is that it may not answer the researcher’s research-related questions or does not contain certain information that the researcher would like to have. Thus, special attention should be given when interpreting secondary data analysis. Further research is needed to validate our models in older cancer survivors at nutritional risks.

Despite these shortcomings, our report is the first study using nationwide data of community-dwelling older cancer survivors who were at risk of malnutrition. We also investigated physical and psychological variables associated with malnutrition using multi-step variable-adjusted models. Based on our results, public health professionals may gain insight on how important it is to detect early malnutrition risk factors and provide timely support in order to relieve individual, social, and worldwide burdens from cancer-related malnutrition.

## 5. Conclusions

Our findings suggest that limited IADL and poor SRH need to be addressed in old cancer survivors who are at nutritional risk. The early recognition and management of malnutrition in these populations might help them to live longer and with a better quality of life, eventually reducing socioeconomic healthcare expenses in our society.

## Figures and Tables

**Table 1:** Differences in individual variables between good and poor nutrition (*n* = 360).

Individual Variables		Classification	Good Nutrition	Poor Nutrition	χ^2^	*p*
			(*n* = 154)	(*n* = 206)		
			*n* (%) or	*n* (%) or		
			M ± SD *	M ± SD *		
Demographic	Age		73.3 ± 5.9	74.6 ± 5.8	2.02	0.045
	Sex	Male	87 (56.5)	93 (45.1)	4.54	0.043
		Female	67 (43.5)	113 (54.9)		
	Marital status	Living with spouse	120 (77.9)	118 (57.3)	16.76	<0.001
		Living without spouse	34 (22.1)	88 (42.7)		
Socio-economic	Education	0–6 years	79 (51.3)	123 (59.7)	3.43	0.331
		7–9 years	29 (18.8)	30 (14.6)		
		10–12 years	32 (20.8)	41 (19.9)		
		≥13 years	14 (9.1)	12 (5.8)		
	Quantiles of household income	Q1 (lowest)	20 (13.0)	47 (22.8)	7.97	0.093
		Q2	32 (20.7)	40 (19.4)		
		Q3	30 (19.5)	46 (22.4)		
		Q4	36 (23.4)	39 (18.9)		
		Q5 (highest)	36 (23.4)	34 (16.5)		
Health status	Disease	Hypertension	60 (39.0)	115 (55.8)	10.03	0.002
		Diabetes	33 (21.4)	61 (29.6)	3.06	0.080
		Arthritis	36 (23.4)	70 (34.0)	4.77	0.029
	BMI **	Underweight (<18.5)	6 (3.9)	17 (8.3)	4.58	0.205
		Normal (≥18.5, <25)	113 (73.4)	132 (64.1)		
		Overweight (≥25)	35 (22.7)	57 (27.6)		
	Number of medication(s)	0	25 (16.2)	8 (3.9)	44.03	<0.001
		1	18 (11.7)	7 (3.4)		
		2	23 (14.9)	10 (4.8)		
		≥3	88 (57.1)	181 (87.9)		
Health-relatedBehavior	Exercise	None	39 (25.3)	68 (33.0)	4.74	0.093
		<150 min. a week	30 (19.5)	48 (23.3)		
		≥150 min. a week	85 (55.2)	90 (43.7)		
	Smoking	Past/Never	8 (5.2)	16 (7.8)	0.94	0.333
		Current	146 (94.8)	190 (92.2)		
	Drinking	None	131 (85.1)	179 (86.9)	4.36	0.113
		≤1 standard drink/day	8 (5.2)	3 (1.4)		
		>1 standard drink/day	15 (9.7)	24 (11.7)		

* M ± SD, mean ± standard deviation. ** BMI, body mass index.

**Table 2:** Differences in physical and psychological characteristics between good and poor nutrition (*n* = 360).

Physical and Psychological Variables		Classification	Good Nutrition	Poor Nutrition	χ^2^	*p*
			(*n* = 154)	(*n* = 206)		
			*n* (%)	*n* (%)		
Physical	Visual discomfort	No	89 (57.8)	121 (58.7)	0.03	0.857
		Yes	65 (42.2)	85 (41.3)		
	Hearing discomfort	No	121 (78.6)	168 (81.6)	0.50	0.482
		Yes	33 (21.4)	38 (18.4)		
	ADL * limitation	No	150 (97.4)	167 (81.1)	22.36	<0.001
		Yes	4 (2.6)	39 (18.9)		
	IADL ** limitation	No	131 (85.1)	117 (56.8)	32.86	<0.001
		Yes	23 (14.9)	89 (43.2)		
	Muscle strength	Good	135 (87.7)	138 (67.0)	20.55	<0.001
		Poor	19 (12.3)	68 (33.0)		
Psychological	Insomnia	No	152 (98.7)	191 (92.7)	7.01	0.008
		Yes	2 (1.3)	15 (7.3)		
	Depression	No	36 (23.4)	91 (44.2)	16.70	<0.001
		Yes	118 (76.6)	115 (55.8)		
	Subjective health status	Positive	76 (49.4)	54 (26.2)	20.45	<0.001
		Negative	78 (50.6)	152 (73.8)		

* ADL, Activities of daily living. ** IADL, Instrumental activities of daily living.

**Table 3:** Multivariable logistic regression analysis of factors associated with nutritional status in elderly cancer survivors.

Variables	Model I	Model II	Model III	Model IV
	OR (95% CI)	OR (95% CI)	OR (95% CI)	OR (95% CI)
Visual discomfort	0.67 (0.41–1.10)	0.68 (0.42–1.12)	0.76 (0.45–1.28)	0.75 (0.45–1.27)
Hearing discomfort	0.65 (0.35–1.20)	0.64 (0.35–1.19)	0.62 (0.32–1.17)	0.62 (0.33–1.19)
ADL * limitation	2.98 (0.90–9.90)	2.90 (0.87–9.65)	2.69 (0.78–9.29)	2.48 (0.72–8.96)
IADL ** limitation	2.13 (1.12–4.06) ***	2.16 (1.13–4.12) ***	2.09 (1.05–4.16) ***	2.15 (1.08–4.28) ***
Poor muscle strength	1.64 (0.85–3.19)	1.64 (0.84–3.18)	1.51 (0.75–3.03)	1.53 (0.75–3.08)
Insomnia	3.94 (0.82–18.88)	3.88 (0.81–18.58)	3.02 (0.62–14.72)	3.09 (0.63–15.02)
Depreesion	1.49 (0.87–2.56)	1.51 (0.87–2.62)	1.60 (0.88–2.87)	1.56 (0.86–2.82)
Negative subjective health	1.84 (1.10–3.07) ***	1.89 (1.13–3.18) ***	1.77 (1.02–3.06) ***	1.74 (1.02–3.02) ***

Model I: adjusted for demographic (age, sex, marital status) characteristics only. Model II: adjusted for demographic and socioeconomic (education and household income) characteristics. Model III: adjusted for demographic, socioeconomic, and health status (disease, BMI, and number of medications) characteristics. Model IV: adjusted for demographic, socioeconomic, health status, and health-related behavior (exercise, smoking, and drinking) characteristics. * ADL, Activities of daily living. ** IADL, Instrumental activities of daily living. *** *p* < 0.05.

## Data Availability

Not applicable.
